# A concise review on factors influencing the hematopoietic stem cell transplantation main outcomes

**DOI:** 10.1002/hsr2.282

**Published:** 2021-05-07

**Authors:** Mohammad Rafiee, Mohammad Abbasi, Hassan Rafieemehr, Amin Mirzaeian, Mohieddin Barzegar, Vahid Amiri, Shaghayegh Shahsavan, Mohammad Hossein Mohammadi

**Affiliations:** ^1^ Department of Hematology and Blood Banking School of Allied Medical Sciences, Shahid Beheshti University of Medical Sciences Tehran Iran; ^2^ Department of Medical Laboratory Sciences School of Paramedicine, Hamadan University of Medical Sciences Hamadan Iran; ^3^ Department of Internal Medicine Hamadan University of Medical Sciences Hamadan Iran; ^4^ Hematopoietic Stem Cells Transplantation Research Center, Laboratory and Blood Banking Department, School of Allied Medical Sciences Shahid Beheshti University of Medical Sciences Tehran Iran; ^5^ Stem Cell Transplantation and Cell Therapy Center Taleghani Hospital Tehran Iran

**Keywords:** engraftment, graft vs host disease, hematopoietic stem cell transplantation, outcomes, relapse, survival

## Abstract

**Background and aims:**

As a curative procedure, hematopoietic stemcell transplantation (HSCT) is an approved treatment for many malignant orbenign hematologic and non‐hematologic diseases. There are different outcomes of HSCT, as well as several parameters influencing these outcomes.

**Methods:**

We had searched scientific sources like Web ofScience and PubMed with a combination of keywords such as HSCT, engraftment,survival, outcomes, etc. Totally, 80 articles were included.

**Results:**

Here we have reviewed the effective factors onmain outcomes of HSCT including engraftment, survival, graft versus hostdisease, and Mobilization. Also, the prediction of hematological reconstitutionand some novel suggestions leading to better outcomes are reviewed.

**Conclusion:**

The study will be applicable for improvedmanagement of autologous and allogeneic HSCT process to increase the procedureefficiency.

## 1 INTRODUCTION

**FIGURE 1:**
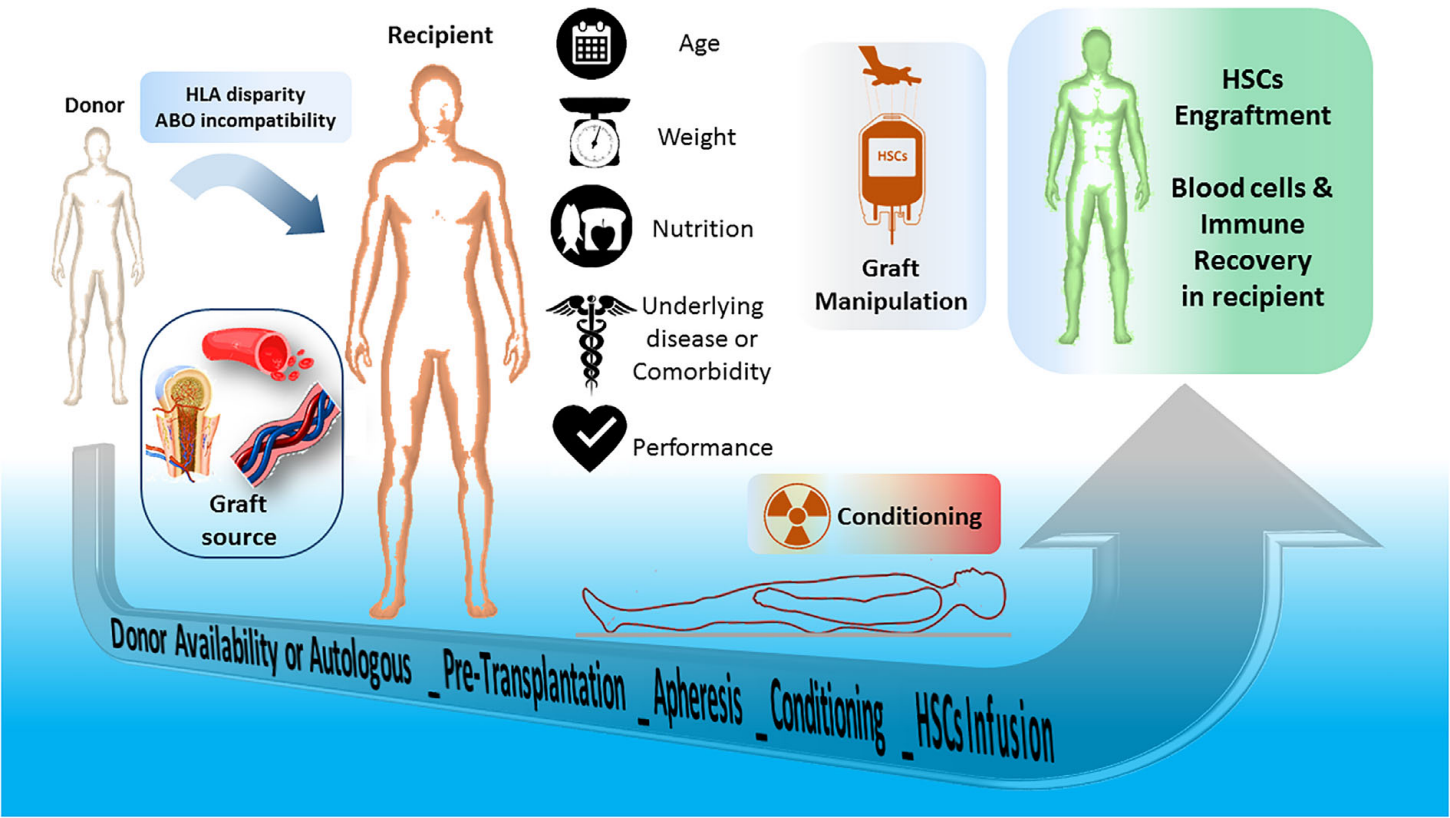
Factors influencing the hematopoietic stem cell transplantation's main outcomes at a glance

Hematopoietic stem cell transplantation (HSCT) is an ever‐evolving field that the attempts for its improvement are still on debate, and, thus far, several strategies such as reduced‐intensity conditioning (RIC) regimens and high‐resolution human leukocyte antigen (HLA) typing have been accomplished to provide a better outcomes.^1^ Table 1 shows the indications for HSCT.^2^ After the infusion of hematopoietic stem cells (HSCs), the competition between donor HSCs and host cells in the bone marrow (BM) microenvironment begins, and the transplantation succeeds when the donor HSCs pass the endothelial barrier to home in the lodgment and then start to proliferate.^3^ With the rapid engraftment post‐HSCT, there would be less demand for blood component infusion, the less incidence of infections, febrile, and bleeding, reduction in the costs due to the less hospitalization duration, and most importantly, survival rates would be improved.^4^ According to the variety of patient populations in transplant centers with different treatment protocols, understanding the effective factors on outcomes is vital.^4^ Although there are several factors, this study concisely reviews the factors influencing the main outcomes of HSCT and discusses the probable approaches, such as modification of conditioning regimens, graft manipulation, and presentation of predictive markers which could result in better outcomes for the HSCT (Figure 1).

**TABLE 1:** Indications of HSCT and percent of HSCTs worldwide

Indications	% of HSCT
Acute myeloid leukemia	33
Acute lymphoblastic leukemia	16
Chronic myeloid leukemia	6
Other leukemia and preleukemia	18
Hodgkin and nonhodgkin lymphoma	12
Multiple myeloma	3
Solid tumor and nonmalignancy	12

## 2 GRAFT vs HOST DISEASE (GVHD)

### 2.1 HLA

**FIGURE 2:**
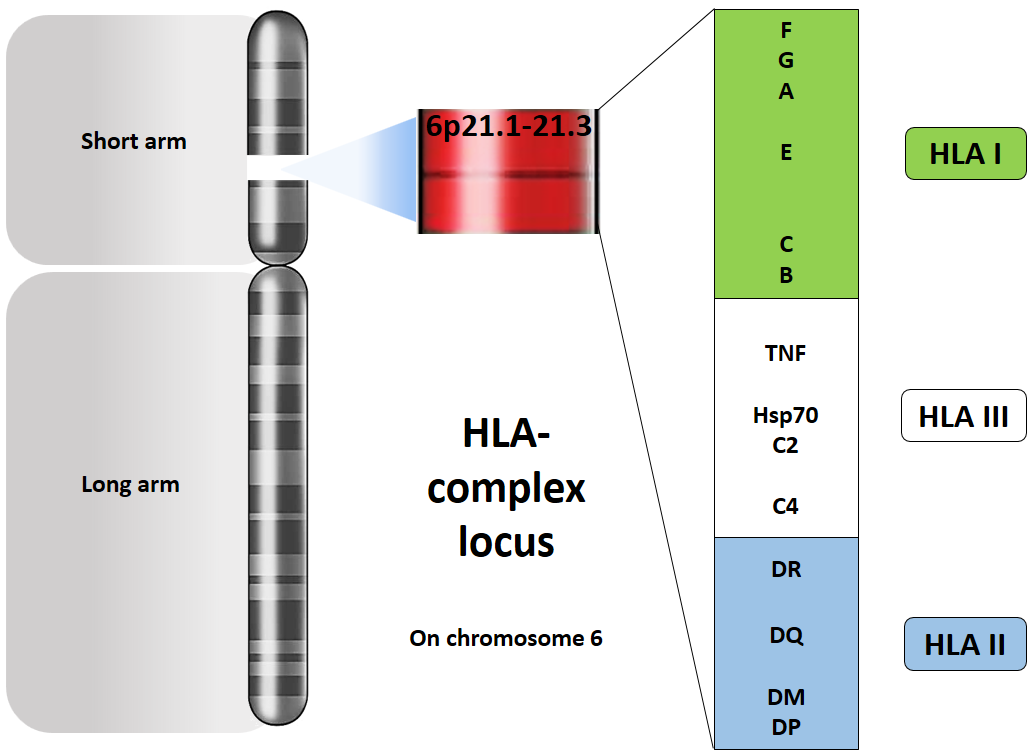
Two important types of HLA in the HSCT are located on the short arm of chromosome 6

The main criterion for donor selection in allogenic HSCT is the HLA compatibility.^5^ Both types of HLA are located on the short arm of chromosome 6 (Figure 2) and are expressed on most of the cells, particularly hematopoietic cells for recognition of foreign antigens.^6^, ^7^ Compatible HLA reduces the risk of GVHD, graft failure, the mortality rate, and increases disease‐free survival (DFS).^7^ Polymorphism of HLA, which is variable from 13 alleles for HLA‐DRB4 to 699 alleles for HLA‐B,^8^ is one of the important limitations for successful HSCT.^7^ Moreover, it has been indicated that the minor incompatibility in allogenic HSCT could be associated with the increased risk of mild GVHD.^9^ Although the best HSCs donors are HLA‐matched sibling or matched unrelated, only less than 30% of patients are lucky enough to have a matched sibling donor (MSD).^10^ After MSD, HLA‐matched unrelated donors (MUD) are the second option, and then the mismatched unrelated donor (MMUD), haploidentical related donor, and umbilical cord blood (UCB) stem cells are alternative donors.^11^

### 2.2 Cell component of grafts

The cell component of grafts is vital about GVHD, as the association between the number of CD3^+^ cells and the incidence of acute and chronic GVHD. It has been reported that high simultaneous doses of CD3^+^ and CD34^+^ cells and high CD3^+^/T regulatory (Treg) ratio in grafts for children with major thalassemia and acute myeloid leukemia (AML) patients increased the rate of acute GVHD.^12^, ^13^ Analysis of cell component along with CD34^+^ cells in grafts is necessary for the risk assessment of GVHD. The incidence of acute GVHD in AML patients is more possible when there is higher number of CD19^+^, CD123^+^, and CD3^+^ cells in grafts. Moreover, the low number of monocytes coupled with high CD34^+^ cells can lead to chronic GVHD.^14^ The grafts cells component can be altered by T‐cell depletion (TCD) as a common graft manipulation to reduce the risk of GVHD. Depletion of total lymphocytes, expansion of lymphocyte Treg, natural killer (NK) cell, and γδ T cells are TCD techniques that could be diminishing the risk of GVHD, and also, immune reconstitution could be improved.^15^ On the other hand, TCD is a potential risk of graft failure,^16^ impairment in immune recovery, and may produce complications like relapse and infections.^15^ Pan T‐cell depletion in HLA mismatch of haplo‐transplantation increased the risk of graft failure and GVHD.^17^ Thus, the TCD method with its advantages and disadvantages should be optimized for the prevention of leukemia relapse, graft failure, GVHD, and infections without the necessity for additional posttransplantation immune supportive actions.^18^

### 2.3 Source of HSCs

Source of HSCs influence the GVHD, for example, although the lowest number of HSCs in the cord blood (CB) compared to BM and the peripheral blood (PB) results in the highest risk for engraftment failure; however, there is the lowest risk of GVHD in this type of HSCT.^10^ Furthermore, the better quality of life (QOL) and well‐being are reported in HLA‐MUD‐HSCT from BM source in comparison with PB‐HSCs; however, the incidence of acute and chronic GVHD has been reported similarly in both cases of HSCT either harvested from BM or PB.^19^

### 2.4 Other factors

There are some other factors that affect the GVHD, like body mass index (BMI) which through the decrease before and after HSCT results in the elevated risk of toxicity by conditioning regimens and GVHD^20^ and the malnutrition that could have negative effects on the outcome of allogenic HSCT through increasing the risk of GVHD and reducing the survival rate of the patients .^21^

## 3 ENGRAFTMENT AND GRAFT FAILURE

As the first outcome of HSCT, the engraftment not only informs the physician about the efficiency of transplantation but also provides a piece of valuable evidence about the later outcomes like sustained hematopoiesis, survival rate, relapse, and the possibility of GVHD.^22^, ^23^, ^24^

### 3.1 HLA and ABO blood group

Some reports suggested that major incompatibility may result in poor graft function, delayed red blood cell (RBC) engraftment, increased risk of graft failure, and shorter overall survival (OS).^25^, ^26^ HLA class I antigens are most important in the determination of engraftment or graft failure.^27^ On contrary, some reports are suggesting that there is no obvious correlation between HLA mismatch and the primary graft failure or the relapse incidence.^1^ The pre‐existing anti‐HLA antibodies in mismatched allogenic HSCT recipients influence the posttransplant chimerism of donor cells, but the existence of these antibodies does not correlate with neither engraftment nor graft failure.^28^

ABO incompatibility has no interfering impact on the neutrophil engraftment, but some certain incompatibilities may result in the posttransplantation pure red cell aplasia (PRCA).^29^ PRCA occurred in 7.5%‐26.1% of HSCT cases with major and bidirectional ABO incompatibility.^30^ A/O (donor/recipient) blood group is an important risk factor for posttransplant PRCA; however, it is reported that transplantation of CB‐HSCs with ABO incompatibility does not result in PRCA.^30^ This is important for the prevention of hemolytic complications and the management of blood bank inventory.^31^ In the cases of HSCT with minor and bidirectional ABO compatibility, there is more necessity for packed RBC (PC‐RBC) units. However, in the cases of major ABO incompatibility, there is more need for platelet units, as compared with ABO compatible donor/recipient .^32^

### 3.2 Disease type and stages

It has been indicated that the engraftment succeeds faster in MM and lymphoma as compared with myelodysplastic syndrome, leukemia, and aplastic anemia.^33^ Graft failure occurs more often in some patients with severe aplastic anemia due to sensitization to RBCs after multiple transfusions, which can be hindered by the reduction of pre‐HSCT transfusion.^27^ Also, in malignant diseases, faster neutrophil engraftment is insignificantly in correlation with the stage of the disease. Due to fewer cycles of chemotherapy and radiotherapy in the early stages of the disease and less damage to the microenvironment of neutrophils precursors in BM, neutrophil engraftment occurs faster than late stages of the disease.^4^

### 3.3 Chemotherapies and conditioning

Availability to BM niche that is occupied by host HSCs is a limitation for engraftment of donor HSCs.^34^ The nonspecific destructive effect of myeloablative conditioning regimens on host HSCs makes BM niches available for infused donor HSCs to engraft.^34^ Given the conditioning regimens, HSCT can be life‐threatening.^35^ Moreover, conditioning not always can eradicate all of the host abnormal cells. Persistence of the host immune system because of RIC or nonmyeloablative conditioning resulted in increased rate of graft failure.^27^ Also, more graft failure is seen in patients without complete remission (CR) before the transplant in comparison to patients with CR.^27^ Currently, the application of monoclonal antibodies like anti‐CD45.2 and anti‐cKit together with conditioning chemotherapy has been suggested to target the immunological barrier for enhancement of engraftment and reduction of toxicity.^35^ The application of anti‐CD45 without chemotherapy or radiotherapy as a conditioning regimen in mice led to the significant increase of donor chimerism. Also, conjugates of anti‐CD45 can target the human primary HSCs and leukemic cell lines *in‐vitro* successfully.^35^

Pre‐HSCT conditioning regimens have an impact on engraftment, and there are several studies which compare the type of the regimens with their effect on engraftment. Busulfan/cyclophosphamide conditioning regimen in autologous HSCT for MM significantly increases the pace of platelet engraftment as compared to high doses of melphalan.^36^ The rate of engraftment after HSCT in AML patients who received fludarabine/low doses total body irradiation (TBI) and cyclophosphamide/fludarabine was faster than those who had high‐dose TBI and cyclophosphamide/TBI.^37^, ^38^ Also, delayed engraftment due to conditioning by thalidomide has been seen in autologous HSCT in MM.^39^ However, Nakasone et al. reported the same engraftment by different doses of TBI as a conditioning regimen in allogenic HSCT.^40^ In another study comparing the various induction therapy for MM (eg, thalidomide‐dexamethasone, vincristine‐adriamycin‐dexamethasone, dexamethasone, lenalidomide‐dexamethasone), no significant difference for neutrophil and platelet engraftment has been reported.^41^ Overall, to achieve personalized medicine in HSCT, attention to the impact of conditioning regimens on the outcome of transplantation is crucial.

### 3.4 CD34^+^ cells value

A viable and adequate number of CD34^+^ HSCs are needed for successful HSCT.^42^ An acceptable number of CD34^+^ cells for achieving the suitable neutrophil and platelet engraftment in HSCT is at least 2 × 10^6^/kg, but it is 2.5 × 10^6^/kg in some studies for optimum engraftment.^4^ Infusion of ≥7.0 × 10^6^ CD34^+^/kg is associated with the significant faster engraftment of platelets.^4^ Dimethyl sulfoxide (DMSO) is used as a cryoprotective of HSCs in the freezing and saving process, but it can damage the HSCs during freeze and thaw. Concerning the importance of CD34^+^ count in graft, a low concentration of DMSO is associated with faster neutrophils and platelets engraftment.^43^

### 3.5 Source and donors

HSCs graft sources may be from BM, PB, and CB. These cell sources are varied regarding their hematologic recovery, graft failure, and GVHD.^44^ Today, the application of BM as a source of HSCs has been decreased, and the significant decrease occurred during 1997‐2006 for patients under the age of 20,^45^ due to the rapid engraftment and hematological recovery of HSCT cases when their HSCs are harvested from PB.^33^ In addition to the invasiveness of harvesting HSCs from BM, the risk of primary graft failure in the transplantation of BM‐HSCs is three times more than that in transplantation of PB‐HSCs.^10^ In the comparison of MUD‐PB‐HSC and double UCB transplantation, neutrophil and platelet recoveries were 13 vs 21.5 days and 19 vs 41 days, respectively.^10^ Graft failure in MMUD is 10% that is higher than MUD, and generally, graft failure in BM source is higher than PB‐HSC (16% vs 3%).^10^ Also, higher engraftment rate and shorter time of engraftment were seen in MSD vs haploidentical donor.^46^

### 3.6 Age, weight, and others

Although HSCT mostly recommended for patients <65 years old^4^ and poor graft function, as a negative outcome of HSCT, was seen in patient >40 years old in allogenic HSCT,^26^ some of the previous studies have shown that age is not a predictive factor for engraftment.^47^ These variations in the results can be due to differences in the patient's condition. Generally, age affects the engraftment and hematological recovery through the total performance of patients. Although it has been suggested that the outcomes of HSCT are better in younger patients, there is no absolute evidence for this statement, and the success of this process depends on several factors. For example, faster engraftment has been occurred in 50‐59 years old in comparison with younger patients.^33^ It has been reported about the weight, as a criterion of performance, that the chance of platelet engraftment was 1.93 times faster when patients weight more than 60 kg.^4^

Viral infections, such as human herpes virus‐6, parvovirus, and cytomegalovirus (CMV), and also the drug use against the infections that can induce myelosuppression (eg, ganciclovir) are associated with graft failure.^24^ It is reported that CMV infection is associated with poor graft function in allogenic HSCT.^26^

### 3.7 Prediction of engraftment

The first outcome of HSCT is the engraftment that is associated with sustained hematopoiesis, GVHD, overall survival, relapse, mortality, morbidity, and QOL.^22^, ^23^, ^24^ Prediction of engraftment is applicable for the risk stratification and management of graft failure, delayed engraftment, and infection. Moreover, this prediction could lead to the early action to modify the pretransplant protocols, such as mobilization, conditioning, management of blood components consumption, hospitalization, and costs.^48^ Today, CD34^+^ cell count per kilogram of the recipient is the only reliable predictive marker for the prediction of HSCT outcome, especially engraftment.^49^ Besides, some other markers or parameters are recently introduced for engraftment prediction, such as colony‐forming unit‐granulocyte macrophage (CFU‐GM) with CD34^+,^ ^48^ subsets of CD34^+^ cell including CD34^+^/CD38^−^, CD34^+^/CD90^−^, and CD34^+^/DR^−^,^50^ immature platelet fraction for platelets engraftment,^51^ and reactive oxygen species (ROS) that accumulate in HSCs during the freeze/thawing process^52^; however, still CD34^+^ cell count is considered to be an important one. The production of ROS in HSCs in the graft of AHSCT has an important influence on neutrophil recovery after transplantation. Accumulation of ROS damages the DNA of CD34^+^ cells.^52^ γH2AX (phosphorylated variant of histone) upregulation is an early response to double‐strand DNA damage.^53^ The calculating ratio of ROS^High^/γH2AX can predict the engraftment time in AHSCT.^52^

## 4 SURVIVAL

### 4.1 HLA

Any mismatches in HLA‐A, ‐B, and ‐DRB1 are associated with the highest mortality.^1^ When there is 10/10 matching in HLA, there is no difference in the OS, DFS, and transplantation‐related mortality (TRM) between HLA‐matched unrelated donor or HLA‐identical sibling donors.^54^ However, matching in HLA‐A and B and HLA‐II alleles is associated with better survival and prevents the incidence of GVHD.^8^ The mortality rate in mismatched HSCT is not only due to the HLA mismatching, but other factors, such as the underlying diseases, could also be responsible. Although HLA‐matched HSCT has the lowest mortality among low‐ and intermediate‐risk patients, there is no evidence of the influence of this factor on the survival rate of the high‐risk patients.^11^ On the other extreme, it is shown that HLA1 antigens mismatch does not associate with OS^55^ and, even in some cases, the result in graft vs leukemia (GVL) as an immune‐mediated phenomenon in allogenic HSCT is a potential curative option for relapsed and refractory Hodgkin and non‐Hodgkin lymphoma.^56^ GVL is a type of controlled GVHD and occurs when HLA‐compatibility is haploidentical.^57^ Control of leukemia without GVHD will be possible by haploidentical‐HSCT.^58^ Nonrelapse mortality in haploidentical‐HSCT is significantly lower than HSCT from MSD, due to the suppressive effect of haploidentical HSCs on recipient neoplastic cells^59^ and thereby result in improved DFS.^60^

### 4.2 Disease type and comorbidities

Despite therapeutic benefits, the success of HSCT is still in the hand of several important risks. For example, certain underlying diseases as co‐morbidities can exacerbate the outcomes of HSCT.^61^ For example, the best OS after HSCT in 1 year was associated with MM,^33^ and HSCT in early ages of sickle cell anemia before the onset of severe organ damage has the best outcome.^62^ Immune‐mediated inflammatory diseases (IMID) are considered to be a prevalent co‐morbidity for HSCT as ulcerative colitis is associated with the highest mortality rate, while rheumatoid arthritis and psoriasis correlate with less mortality among IMIDs.^63^ Thus co‐morbidity index (CI) and disease risk index (DRI) should be measured as predictive parameters for predicting the outcome and OS after HSCT.^61^

### 4.3 Weight, nutrition, and exercise

Higher nonrelapse mortality (NRM) and lower OS in underweighted and increased NRM in the obese HSCT candidates were reported. Higher NRM in obese patients can be due to the higher intensity of conditioning regimens in comparison with normal‐weighted patients.^64^ Nutritional status by quantification of albumin and BMI is generally used for evaluation of overall health, and it has been reported that pretransplant BMI < 18.5 kg/m^2^ is associated with the higher risk of relapse, the TRM, as well as the lower survival.^20^ Regarding the progressive increase in adipose and decrease in muscular tissue during adulthood and the elderly, patients at nutritional risk or poor nutrition status should be recognized, that the best chemotherapy regimens would be decided for them, as these patients are at the risk of chemotherapy‐related toxicity.^65^ The nutritional and body composition profile should be analyzed for the elderly patient before and after HSCT for managing their treatment and hospitality.^65^ Exercise, as supportive care for HSCT patients, has a significant benefit for physical performance and results in the rapid recovery of the immune system and reducing the side effect of the therapy.^66^ Furthermore, improvement of QOL, especially emotional aspects, is attributed to exercising before and after HSCT.^66^

## 5 MOBILIZATION (AS AN OUTCOME)

There are some mobilizer drugs with a different mechanism like granulocyte‐colony stimulating factor (G‐CSF) and plerixafor. Through the expansion of the myelo‐monocytic series, G‐CSF activates the proteolytic enzyme, which in turn by breaking the receptor‐ligand bonds between HSCs and BM niche cells increased the release of HSCs to PB.^67^ CXCR4 as a chemokine receptor on HSCs bind to stromal cell–derived factor 1α (SDF‐1α) and preserve these cells in BM niches. Plerixafor separates the CXCR4‐SDF‐1α interaction by reversibly binding to CXCR4 and results in mobilization of HSCs to PB..^68^ Unlike several advantages in the harvest of HSCs from PB, such as being less invasive, no need for anesthesia, and more rapid engraftment of infused PBSCs,^16^ poor mobilization is one of the fundamental problems in autologous HSCT which has been reported in 3%‐46% of autologous HSCT cases.^69^, ^70^ Prolonged time of apheresis, alternative mobilizer drugs, and increased time of mobilizer drugs are the compensatory solutions for poor mobilization.^71^ The combination of G‐CSF and plerixafor results in more HSCs mobilization, and there are no significant side effects as compared to G‐CSF, as a single agent.^68^ Yuan et al, have shown that plerixafor could have earlier engraftment than G‐CSF; nonetheless, no difference in long‐term outcome of HSCTs has been reported between these two mobilizer drugs.^72^ Furthermore, some effective parameters should be considered to optimize the mobilization. Circadian rhythms govern the count of HSCs in peripheral blood, and acute physiological stress like exercise also mobilizes the HSCs to circulation.^71^ Moreover, it has been suggested that the combination of plerixafor with a single dose of Viagra, used orally, could increase the HSCs mobilization through changing vascular integrity and trafficking of HSCs.^73^ Minimum acceptable number of mobilized CD34^+^ HSCs is 2 × 10^6^/kg of recipients weight and 5 × 10^6^/kg considered to be adequate for autologous HSCT.^74^

## 6 A NEW METHOD FOR IMPROVEMENT OF OUTCOMES

Mesenchymal stem/stromal cells (MSCs), such as fibroblast‐like cells, provide a specialized microenvironment for HSCs in BM by secretion of cytokines, growth factors, extracellular matrix, and extracellular vesicles (EVs), which are vital for hematopoietic stem cell differentiation, proliferation, and maintenance.^75^, ^76^ Secretion of stem cell factor (CXCl12) for maintenance and protection of HSCs is the main function of MSCs. Also, the downregulation of leptin receptor (LepR), expressed on MSCs, results in the reduction of quiescent HSCs in BM.^77^ In HSCT context, MSCs utilize two main methods: co‐culture of MSCs with HSCs before transplantation and co‐administration of MSCs with HSCs in infusion to the recipient in phase I/II clinical trials and animal models that cause rapid reconstitution and lower toxicity and graft failure.^77^, ^78^ Noteworthy, co‐administration of MSCs with allogenic haplotype HSCT prevents the GVHD.^79^

Today, extracellular vesicles (EVs) are known as a new mediator of the cell‐to‐cell communication. These nano‐sized vesicles mimic the parental cells (eg, MSCs) by transferring their content including protein, mRNA, DNA, microRNA, and organelles and exert their function on target cells by merging into the membranes.^80^, ^81^ It is now clear that EVs in BM play important roles in the physiological niche (eg, cell survival, proliferation, differentiation, and angiogenesis) and malignant niches (eg, tumor progression, chemo‐resistance, immunosuppression).^82^ Recent studies suggested the use of MSCs‐derived EVs (MSC‐EVs) for the improvement of Allo‐HSCT (as a graft manipulation). It is demonstrated that exposure of UCB‐CD34^+^ HSCs to MSC‐EVs increases the viability, reduces the differentiation, and up‐regulates CXCR4 (with function in homing) in UCB‐HSCs.^83^ Also, miRNA and piwi‐RNA from BM‐MSC‐EVs induce survival and inhibit differentiation in UCB‐HSC, which is considerable in transplantation.^79^ Stability of MSC‐EV content and fewer side effects are advantages of EVs application vs soluble content and MSCs.^83^

## 7 CONCLUSION

The importance of the identification of the effective factors on the outcome of autologous and allogenic HSCT is well‐established in several reports as there are some links between these factors and the elevated risk of some complications, such as infections, relapse, and GVHD post HSCT. Apart from being a duty of transplantation centers, previous awareness about engraftment, complications, and other outcomes is necessary to achieve the optimized costs and the amount of blood components consumption. This study was reviewed for the main outcomes and their known influencing factors and emphasizes the importance of further studies for discovering more reliable predictive factors for HSCT outcomes.

## CONFLICT OF INTEREST

The authors declare no conflict of interest.

## TRANSPARENCY STATEMENT

The lead author affirms that this manuscript is an honest, accurate, and transparent account of the study being reported; that no important aspects of the study have been omitted; and that any discrepancies from the study as planned have been explained.

## AUTHORS CONTRIBUTION

Conseptualization: Mohammad Rafiee, Mohammad Abbasi, Hassan Rafieemehr.

Investigation: Mohammad Rafiee, Vahid Amiri, Mohieddin Barzegar.

Supervision: Mohammad Rafiee.

Validation: Mohammad Rafiee, Mohammad Abbasi, Mohammad Hossein Mohammadi.

Visualiation: Mohammad Rafiee.

Writing ‐ Original Draft Preparation: Amin Miraeian, Vahid Amiri, Mohieddin Baregar, Shaghayegh Shahsavan.

Writing ‐ Review & Editing: Mohammad Rafiee, Mohammad Hossein Mohammadi.

All authors have read and approved the final version of the manuscript.

Mohammad hossein Mohammadi had full access to all of the data in this study and takes complete responsibility for the integrity of the data and the accuracy of the data analysis.

## Data Availability

The data that are reviewed in this study are available in scientific sources (with their citation in references).
